# De-psychiatrizing our own research work

**DOI:** 10.3389/fsoc.2022.929056

**Published:** 2022-08-11

**Authors:** Jasna Russo

**Affiliations:** Department of Social Work, Alice Salomon University of Applied Sciences, Berlin, Germany

**Keywords:** de-psychiatrization, knowledge-production, co-optation, research labor, first-person knowledge

## Introduction

In this brief opinion piece, I focus on the processes of psychiatrization of one's own work. Regardless of our intentions, what we do can, in the long term, ultimately enforce the very phenomenon that we seek to expose and disrupt. I challenge the belief that the psychiatrization of society can be interrogated from any knowledge-making site that is itself safe from psychiatrization, and hope to re-direct the analysis from a rather general notion of “society” toward our own work. Adopting Oliver's understanding of research as social production (1992), I engage with our own responsibility for the ways in which we design and conduct inquiries, and the potential of the research process itself to ultimately replicate or transform the status quo.

## Do we need further proof of the psychiatrization of society?

Beeker et al. (2021) call for transdisciplinary research to “empirically prove that psychiatrization exists, developing valid indicators for its extent” (p. 8) and to “empirically assess causes, mechanisms and effects of psychiatrization” (p. 1). Undeniably, it is important to understand how psychiatrization occurs, particularly given its ever subtler modes of operation. However, taking into account the history of psychiatry, it is hard to comprehend the requirement to prove that “[o]n a societal level, psychiatrization *might* boost medical interventions which incite individual coping with social problems, instead of encouraging long-term political solutions” (Beeker et al., 2021, emphasis added). A considerable body of scholarship has already demonstrated that the individualization and medicalisation of social problems are at the heart of psychiatry (see for example Foucault, 1973; Conrad, 1992; Burstow, 2015—to mention just a few). Various thinkers (e.g., Foucault, 1977; Kanani, 2011; Joseph, 2015) have traced psychiatry's historical role in upholding regimes of oppression on the grounds of their “medical” justification. The repeated psychiatrization and subsequent de-psychiatrization of particular lives goes hand in hand with broader social changes. This trend can be observed in the establishment and subsequent abandonment of particular diagnoses, from drapetomania^1^ and dysaesthesia aethiopica^2^ to homosexuality and transsexualism. The psychiatrization of society can be understood as integral to psychiatry's purpose and social function. Rather than signifying a contemporary development yet to be empirically proven, this process goes back to the creation of first psychiatric institutions and the formation of psychiatry as a medical discipline. Acknowledging the psychiatrization of society as factual, rather than hypothetical, could straightforwardly direct research efforts toward de-psychiatrization. However, the requirement to provide *evidence* of the lived reality and experiential knowledge of many typifies the “slowness of science and urgency of need” (Russo and Stastny, 2009). This approach to knowledge production obscures the need to act upon the already available evidence, and has far-reaching consequences in maintaining the status quo. Commitment to de-psychiatrization, though, requires a shift “from needing more knowledge to needing values that allow us to take a stand with respect to what we know” (Frank, 2000, p. 363).

## Reducing psychiatrization vs. de-psychiatrization

The release of DSM-5 (American Psychiatric Association, DSM-5 Task Force, 2013) raised many concerns “that clients *and the general public* are negatively affected by the continued and continuous medicalization of their natural and normal responses to their experiences” (British Psychological Society, 2011, emphasis added). The call to put this development on the official research agenda comes after a sufficiently large population is affected (American Psychological Association Division 32, 2011) and hardly anybody in the Western world can be safe from receiving a psychiatric diagnosis and treatment. Furthermore, the adoption of the Convention on Rights of Persons with Disabilities (United Nations, 2007) has ushered in an era characterized by the introduction of human rights discourse in a heretofore exclusively medical realm. This climate sharpens antagonisms within the psychiatric establishment (see the debate in World Psychiatry, 2019) and gives rise to in-house initiatives to humanize psychiatry. However, there is a significant difference between long-term efforts to scrutinize and challenge psychiatrization as a dominant social response to madness and distress (e.g., Szasz, 1961; Burstow, 2015; Russo and Sweeney, 2016) on one side and attempts to limit its scope to a reasonable number of “severely ill cases” on the other. The latter approach is exemplified in the following statement by Beeker et al. (2021):

“While individuals with minor disturbances of well-being might be subjected to overdiagnosis and overtreatment, psychiatrization could also result in undermining mental healthcare provision for the most severely ill by promoting the adaption of services to the needs and desires of the rather mild cases.”

The contested presumption of the biomedical nature of madness and distress remains implicit in the work of many critical psychiatry scholars who seek to engage in “less psychiatrizing forms of psychosocial support” (von Peter et al., 2021) or routinely assume the existence of “apolitical or irreducible distress” (Logan and Karter, 2022). This kind of subtle but persistent othering subverts efforts to eradicate the psychiatrization of human experience as a matter of principle (LeFrançois et al., 2013; Burstow et al., 2014; Russo and Sweeney, 2016; Beresford and Russo, 2021), regardless of its spread—and despite circumstances that can turn “mental illness” into an acceptable explanatory framework that legitimizes medical “solutions” to the complexities of living. The latter trend is acknowledged as “bottom up psychiatrization” (Beeker et al., 2021; Logan and Karter, 2022) but overlooks the many intersections of knowledge-making processes in which top-down and bottom-up psychiatrization merge. Given that knowledge-production takes place at precisely these junctures, it is crucial to illuminate that blank space and render the research labor visible. The failure to even position ourselves and our own research work in relation to what we study is still wide-spread, and is paradigmatic to dominant understandings of research. In the case of psychiatrization (see Figure 1 provided by Beeker et al., 2021), this kind of detachment suggests that we, as researchers, have little or no role in the processes we are supposed to investigate. In reality, however, our inquires do not occur at some safe, distant remove from, but rather from the midst of psychiatrized societies' institutions, their dominant paradigms and related criteria of what constitutes evidence. We cannot explore the psychiatrization of society without looking at our role as knowledge-producers and, most importantly, considering our own agency and responsibility for the contributions we make.

## The vicious circle of official knowledge-making on madness and distress

Regardless of our own theoretical, methodological, personal or political backgrounds, taking part in the official production of knowledge on madness and distress inevitably means entering a realm dominated by the biomedical conceptualization of “mental illness.” The discipline of psychiatry not only maintains definitional power in terms of the identities, treatments and ideology it produces, but also forces all official knowledge-making into an ongoing dialogue with the biomedical model. Attempts to establish alternatives to this model are subjected to this same process: in order to prove eligible and fundable at officially recognized knowledge-making sites, such projects need to satisfy the current criteria of what constitutes scientific validity and evidence base. The psychiatrization of society is therefore inseparable from the psychiatrization of knowledge-production on madness and distress—or, in the words of the UK long-term survivor activist Campbell (1996):

“Psychiatry would see itself as the servant of society. Yet it is naive to suppose that a profession with such an individual and collective power does not *form* as well as reflect public attitudes. If we think of emotional distress as mental illness it is psychiatry that has seduced us so.” (p.57, emphasis added).

The fundamental challenge faced by researchers committed to working against, or despite, the dominant paradigm is how to break the self-perpetuating mechanism that, in the end, annexes and psychiatrizes all advancements in knowledge, including practices and epistemologies that have nothing to do with the biomedical approach to begin with. Here I particularly mean research, theoretical concepts and a variety of collective, non-medical, self-organized responses to madness and distress, developed by individuals and organizations of people who have been on the receiving end of psychiatric “care.” Personally, I am more familiar with the developments in highly psychiatrized Western societies, but similar processes of co-optation can be seen in the movement for global mental health that targets countries of the Global South (Logan and Karter, 2022) and “merges psychiatric knowledge with the idea of a ‘social movement”' (Fey and Mills, 2021:193). Survivor research (Sweeney, 2016a,b) and other work that explicitly aims at de-psychiatrization—informed by our experiences and knowledge gained through the de-psychiatrization of our own biographies – continues to be selectively employed to extend and supplement the biomedical paradigm with “lived-experience” perspectives. The low status of first-person knowledge, combined with extremely unequal distribution of resources, renders our efforts susceptible to re-psychiatrization (Costa et al., 2012; Penney and Prescott, 2016; Russo, 2016). In his excellent analysis of how emancipatory ideas and practices dissolve and can subsequently turn into their opposite, Fabris (2016) highlights that even “writing as a form of protest can easily be usurped by the systems seeking ‘newness”' (p. 99) and reminds us that community treatment orders were “once a rosy deinstitutional notion” (p. 97). Other, similar developments include the insertion of “peer specialists” into psychiatric practice (Davidow, 2013; Brown and Stastny, 2016) and “service user involvement” in mental health research (Staddon, 2013). How, then, are we supposed to work for change while being aware of the system's need and power to co-opt? This is a complex question that calls for a variety of individual answers and context-specific strategies, rather than any universal solution. How to actually enact transformative research within, beside or outside of the existing structures of knowledge production is a whole different issue, worth exploring on its own. Turning to areas outside of those with which we are familiar, and in which we often feel stuck, can help us understand some common patterns and develop new perspectives. In his seminal work on the structures of scientific revolutions, Kuhn (1996) explores how paradigm shifts actually occur. He suggests “that there are excellent reasons why revolutions have proved to be so nearly invisible” and that most of them “have customarily been viewed not as revolutions but as additions to scientific knowledge” (p. 136). Accepting this course as logical and unavoidable might help us re-examine our understanding of what constitutes success, and persist in our efforts despite not seeing tangible changes in the way we expect to see them.

## Emancipating research labor

In this brief opinion piece, I have criticized the framing of the psychiatrization of society as a contemporary development and suggested that the release of the DSM-5 (2013) marks just one of the pinnacles of that process, rather than its beginning. I point to the role of knowledge-production in the psychiatrization of society and argue for a straightforward shift toward de-psychiatrization. What remains impossible to provide is any general answer to the question of how to prevent (re)psychiatrization of one's own research work. I hope for future debates, alliances and action around this crucial issue.

It is clear that we cannot determine the long-term journeys of the outputs we create and the many ways in which our work can be utilized. But there are important aspects that we can influence—from ensuring the ethics, quality, and the transformative power of the research process itself, to prioritizing our audiences and determining how we communicate our work. The disability researcher and theoretician Oliver (1992, 2009) approaches research as a form of social production and understands it not as “attempt to change the world through the process of investigation but an attempt to change the world by producing ourselves and others in differing ways from those we have produced before” (2009, p. 116). Adopting this view brings us back to our own work and our responsibility for what we create within the radius of our own projects, no matter how limited that radius might appear. De-psychiatrizing our own research is therefore both a personal issue and a matter of politics and strategy. Although I am convinced that there are ways to emancipate our approaches from the (retrograde) currencies of our respective disciplinary fields, such emancipation might not be possible from within the particular field of psychiatry, for the reasons outlined above.

## Author contributions

The author confirms being the sole contributor of this work and has approved it for publication.

## Conflict of interest

The author declares that the research was conducted in the absence of any commercial or financial relationships that could be construed as a potential conflict of interest.

## Publisher's note

All claims expressed in this article are solely those of the authors and do not necessarily represent those of their affiliated organizations, or those of the publisher, the editors and the reviewers. Any product that may be evaluated in this article, or claim that may be made by its manufacturer, is not guaranteed or endorsed by the publisher.

## Footnotes

^1^*Enslaved Africans Fleeing Captivity*. Available online at: https://en.wikipedia.org/wiki/Drapetomania.

^2^*Laziness Among Slaves*. Available online at: https://en.wikipedia.org/wiki/Dysaesthesia_aethiopica.
